# Infantile *ATP7B*-Related End-Stage Liver Disease: An Exceptional Wilson Disease Phenotype From Consecutive Generations

**DOI:** 10.1097/PG9.0000000000000112

**Published:** 2021-08-05

**Authors:** Emanuele Nicastro, Maria Iascone, Angelo Di Giorgio, Jernej Brecelj, Raffaella Petruzzelli, Roman S. Polishchuk, Maesha Deheragoda, Bart E. Wagner, Aurelio Sonzogni, Ezio Bonanomi, Lorenzo D’Antiga

**Affiliations:** From the * Pediatric Hepatology, Gastroenterology and Transplantation, Hospital Papa Giovanni XXIII, Bergamo, Italy; † Medical Genetics Laboratory, Hospital Papa Giovanni XXIII, Bergamo, Italy; ‡ Departments of Gastroenterology, Hepatology and Nutrition and Pediatrics, Medical Faculty Ljubljana, University Medical Centre Ljubljana, Ljubljana, Slovenia; § Telethon Institute of Genetics and Medicine (TIGEM), Pozzuoli, Italy; ‖ Institute of Liver Studies, King’s College Hospital, London, United Kingdom; ¶ Histopathology Department, Royal Hallamshire Hospital, Sheffield, United Kingdom; # Pathology, Hospital Papa Giovanni XXIII, Bergamo, Italy; ** Pediatric Intensive Care Unit, Hospital Papa Giovanni XXIII, Bergamo, Italy.

**Keywords:** neonatal cholestasis, consecutive generations, copper, liver transplantation, next generation sequencing

## Abstract

Wilson disease (WD) is a rare autosomal recessive disorder of copper metabolism typically presenting after 3 years of age. We describe a girl presenting with neonatal cholestasis rapidly progressing to end-stage liver disease. She presented hepatosplenomegaly, neurological impairment, Coombs-negative hemolytic anemia, central hypothyroidism. A patient-parents whole exome sequencing identified a homozygous state for *ATP7B* mutations causing WD in the proband (p.Gln7fs/p.His1069Gln) and her mother (p.His1069Gln/p.His1069Gln), who was then confirmed to have cirrhotic WD. A causative role of copper toxicity due to *ATP7B* loss of function was indicated by the presence of extrahepatic features of WD, consistent tests of copper metabolism—including a 7-fold increase in liver copper—and similarity of patient’s liver gene expression profile and ultrastructure with that of WD models. This exceptionally early presentation could result from the combination of the *ATP7B* impairment and the intrauterine copper overload due to maternal undiagnosed WD.

## Abstract 2

Supplemental Digital Content is available in the text.

## INTRODUCTION

Wilson disease (WD) is an autosomal recessive disorder caused by mutations in the *ATP7B* gene, encoding a copper-transporting adenosine triphosphatase responsible for the ion excess handling and biliary excretion. Copper accumulation and toxicity cause liver disease and multiorgan injury, typically presenting after 3 years of age (1).

We describe a girl with neonatal cholestasis progressed to cirrhosis and end-stage liver disease. Child-parents urgent whole exome sequencing (WES) revealed *ATP7B* biallelic mutations in the child and her mother, supporting the concomitant diagnosis of WD in consecutive generations. We provide clinical, biochemical, and proteomic data supporting the pathogenic role of *ATP7B* impairment in this patient, possibly in addition with the prenatal copper overload.

## CASE REPORT

**FIGURE 1.**
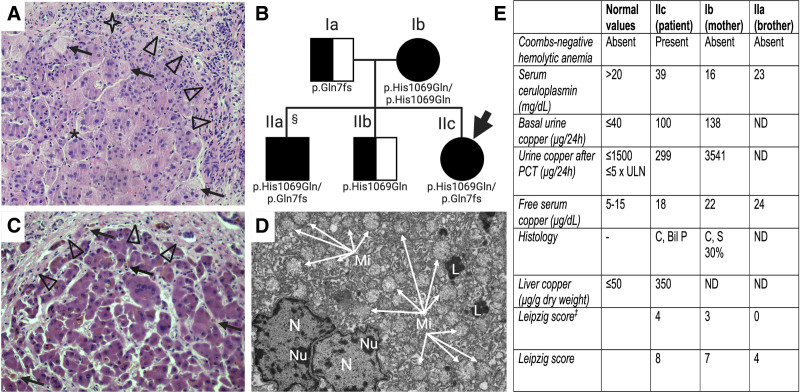
Clinical, biochemical, and histologic features of the patient. A) Liver histology at 2 mo of life, showing diffuse giant cell transformation, fibrous septa (arrowheads), microsteatosis (*), feathery degeneration of the hepatocytes (arrows), minimal portal infiltrate (star). B) Pedigree chart of the patient (arrow) showing *ATP7B* genotype; the patient’s older brother (§) was a presymptomatic subject diagnosed with WD on familial screening. C) Liver histology on hepatectomy specimen, showing cirrhosis (arrowheads) and biliary plugs (arrows). D) Transmission electron microscopy of hepatectomy specimen. Hepatocytes show increase in number of mitochondria (arrows) with artifactual swelling. E) Tests of copper metabolism in the patient (IIc) and her mother (Ib) and brother (IIa). Leipzig score 0–1: WD unlikely; 2–3: WD probable; ≥4: WD highly likely; ‡score calculated without including the *ATP7B* genetic testing. Bil P = biliary plugs; C = cirrhosis; L = secondary lysosomes; Mi = mitochondria; N = nucleus; ND = not done; Nu = nucleolus; PCT = penicillamine challenge test; S = steatosis; WD = Wilson disease.

A Bosnian female infant born to nonconsanguineous parents presented with jaundice in ABO incompatibility, and developed cholestasis with hepatosplenomegaly. Family and pregnancy history were unremarkable. She had normal gamma-glutamyltranspeptidase, high serum bile acids, aspartate aminotransferase > alanine aminotransferase. A liver biopsy at 2 months of age showed diffuse giant cell transformation, canalicular cholestasis, moderate lobular activity, minimal steatosis, extramedullary hematopoiesis (Fig. 1A). She was started on ursodeoxycholic acid 20 mg/kg/d.

She worsened, and was evaluated for LT at 7 months with total/conjugated bilirubin of 22/20 mg/dL, prothrombin time international normalized ratio 1.9, aspartate aminotransferase/alanine aminotransferase ×4/×1.5 upper limit of normal, normal gamma-glutamyltranspeptidase, ascites and prerenal kidney injury. She was admitted to pediatric intensive care with multiorgan failure, severe neurological impairment (hypertone, tremor, poor spontaneous motility), and mild left ventricular myocardial hypertrophy. Laryngotracheomalacia, Coombs-negative hemolytic anemia, and central hypothyroidism were also present. After surgical closure of patent ductus arteriosus, she was listed for LT (pediatric end-stage liver disease score = 34). An extensive work-up ruled out infectious and metabolic etiologies (Table 1). An urgent child-parents WES revealed that the girl was compound heterozygote for 2 known WD causing mutations in the *ATP7B* gene (c.19-20del, causing p.Gln7fs, and c.3207C>A, causing p.His1069Gln); the mother was homozygote for p.His1069Gln, and then was confirmed having WD with cirrhosis, while the father carried p.Gln7fs (Fig. 1B). Patient’s copper metabolism revealed normal serum ceruloplasmin (39 mg/dL, NV > 20), increased basal cupruria (100 μg/24 h, NV < 40), and free cupremia (Fig. 1E). The brain magnetic resonance showed unspecific brain injury: focal (left occipital and periventricular) T2 hyperintensity suggesting gliosis, along with mild supratentorial white matter atrophy, while basal ganglia were normal. She was started on penicillamine 20 mg/kg/d and transplanted soon thereafter. Liver copper on hepatectomy was 350 μg/g dry weight (>5 × upper limit of normal). Histology showed cirrhosis and biliary plugs (Fig. 1C). Hepatocyte ultrastructure showed increased number of mitochondria (Fig. 1D).

**TABLE 1.** Biochemical investigation protocol performed in the patient

Endocrine work-up	Cortisol, ACTH, TSH, and thyroxine
Virologic work-up	Serology (IgG and IgM): HAV, HBV, HCV, HEV, Epstein-Barr Virus (EBV, EBNA IgG, and EBV-VCA IgM), CMV, HSV-1/2, Toxoplasma gondii, rubella, parvovirus B19
	Nucleic acids: EBV-DNA, CMV-DNA, HSV-1/2-DNA, human herpes virus 6
Metabolic work-up	Serum alpha-1 antitrypsin, plasma and urine amino acids, urine organic acids, serum total bile acids + mass spectrometry urine analysis, transferrin isoelectric focusing, acylcarnitine profile, pyruvic acid, lactic acid, erythrocyte galactose-1-phosphate uridyl transferase activity, urine galactose, urine fructose, chloride sweat test, beta-glucosydase, lysosomal acid lipase, and acid sphingomyelinase on dried blood spot mass spectrometry

ACTH = adrenocorticotropic hormone; CMV = cytomegalovirus; EBNA = Epstein-Barr nuclear antigen; EBV = Epstein-Barr Virus; HAV = hepatitis A; HBV = hepatitis B; HCV = hepatitis C; HEV = hepatitis E; HSV-1/2 = herpes simplex virus-1 and-2; TSH, thyroid-stimulating hormone; VCA = viral capsid antigen.

She is alive and well 4 years after LT with complete neurologic recovery. The mother is well under penicillamine. A 7-year-old brother was diagnosed with asymptomatic WD by screening and started on zinc acetate.

**FIGURE 2.**
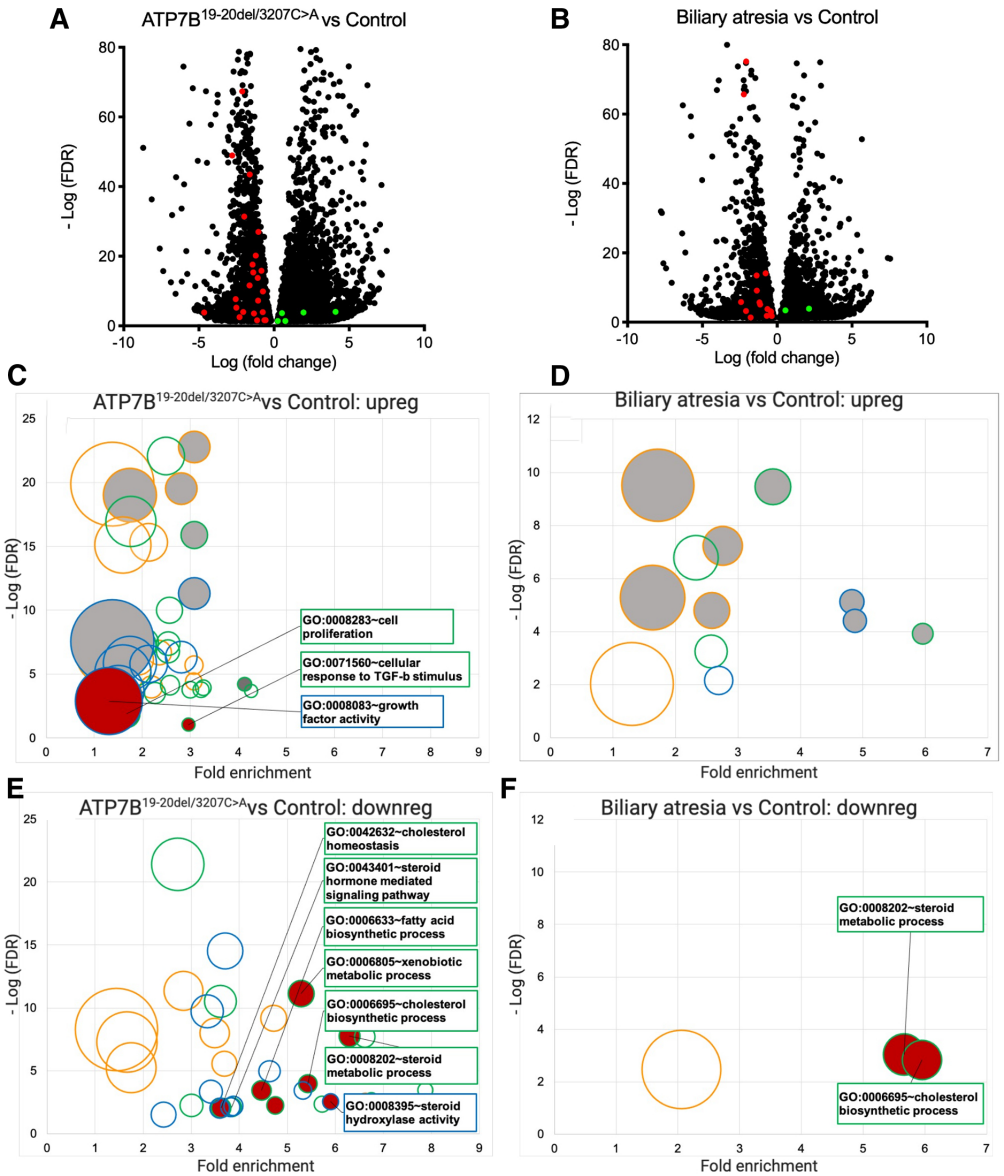
Gene expression studies. A, B) Volcano plots showing statistically significant changes in gene expression in liver tissue at hepatectomy of the patient (A) and in 3 liver specimens at hepatectomy from patients with biliary atresia (B). On the x-axis, the log_10_ of the fold change gene expression with regard to a healthy donor liver is displayed; On the y-axis, data are plotted according to the negative log_10_ of the FDR, expressing the statistical significance of the change; the colored spots indicate the significantly up- (green) or downregulated (red) Wilson disease–related genes. C–F) Bubble plot of gene ontology enrichment analysis of statistically significant gene subsets up- or downregulation in liver tissue at hepatectomy of the case patient (C, upregulated; E, downregulated) and in 3 liver specimens at hepatectomy from patients with biliary atresia (B, upregulated; D, downregulated). On the x-axis, the log_10_ of the fold enrichment (expressing the representativeness of the gene subset) with regard to a healthy donor liver tissue is displayed. On the y-axis, data are plotted according to the negative log_10_ of the FDR, expressing the statistical significance of the change; gene subsets are classified as biological processes (green bubbles), cell component (orange bubbles), and molecular function (blue bubbles); bubble size is proportional to the number of gene included in the subset. The red-filled bubbles are the gene subsets up- or downregulated in gene ontology analysis in Wilson disease rodent models (2). The grey-filled bubbles are gene subsets related to liver fibrosis. FDR = false discovery rate.

The liver differential transcription pattern (fold change from a healthy liver) of the patient showed higher similarity to WD than that of 3 biliary atresia (BA) control patients: WD-related up- or downregulated genes were 28/61 versus 18/61 in the patient and in BA controls, respectively (Fig. 2A, B).

In both patient and BA livers, an upregulation of gene clusters related to the extracellular matrix organization was present, due to fibrosis (Fig. 2C, D). However, the patient had higher similarity with WD as for an upregulation of the gene subsets related to cell proliferation and response to stimuli, and downregulation of those related to sterol, fatty acid, and cholesterol metabolism and xenobiotic metabolic processes (Fig. 2C–F). Methods for genetic testing (WES), pathology studies, and RNA studies are described in the Supplemental Digital Content, http://links.lww.com/PG9/A58.

## DISCUSSION

Reduced fertility and spontaneous miscarriages are common in WD-untreated mothers, and anecdotal descriptions of copper accumulation in the placenta and in live birth fetuses also exist (3).

This is the first report of a severe infantile liver disease possibly due to WD causing *ATP7B* mutations. Intrauterine copper exposure due to maternal undiagnosed WD might have contributed to this exceptional phenotype.

WD typically present after infancy, possibly as the result of the *ATP7B*-independent embryo-fetal and neonatal copper metabolism. At birth, the ion is mostly accumulated intracellularly bound with metallothioneins, while its mobilization toward mitochondria, binding to ceruloplasmin, and ATP7B-driven biliary excretion occur later in infancy (4).

Prenatally accumulated copper may have caused toxicity in the *ATP7B*-deficient liver when ion handling became *ATP7B*-dependent.

In support of this hypothesis, the patient showed clinical extrahepatic aspects of WD, met the Leipzig diagnostic criteria including a 7-fold increase in liver tissue copper content, and showed gene expression profile consistent with that of available animal models of WD.

The biochemical markers of copper metabolism should be interpreted with caution in an infant. Liver copper content can be higher than the normal in non-WD cholestatic liver diseases and in healthy newborn and infants (5). Also, the normal serum ceruloplasmin concentration of the present case could be explained by the acute-phase response typical of neonatal/infantile hepatitis. Nevertheless, WD patients carrying at least one missense mutation (like the proband and her brother) are described to have higher and even normal levels of ceruloplasmin (6). The initial histological changes—predominated by hepatocyte giant cell transformation—reflect the most frequent and stereotyped pattern of liver injury in infants. On the other hand, the ultrastructural picture of an expanded mitochondrial compartment could fit the hypothesis of the copper toxicity but also be related to anoxia due to delay in fixation, which is common in hepatectomy specimens. Age-dependent copper accumulation in the mother might explain the fact that—7 years earlier—a pregnancy with the same *ATP7B* fetal genotype did not present with the same phenotype. However, also genes other than *ATP7B* as well as epigenetic factors are known to play a role as phenotype modifiers (7).

Given the limits of a single case description, the causative role of *ATP7B* mutations in determining severe infantile cholestatic liver disease needs to be confirmed in similar cases.

## Supplementary Material


