# Supplementary material for: Infantile ATP7B-Related End-Stage Liver Disease: An Exceptional Wilson Disease Phenotype From Consecutive Generations
Source: JPGN Rep. 2021 Aug 5;2(3):e112. doi: 10.1097/PG9.0000000000000112 (PMC10191499; doi:10.1097/PG9.0000000000000112)
Supplement: Supplementary file 1 [file pg9-2-e112-s001.pdf]

METHODS

Genetic testing

WES was performed using the SureSelect Clinical Research Exome v2.0 enrichment kit (Agilent Technologies, Santa Clara, CA). Variant annotation and filtering were performed as previously described [(1)]. Variants were filtered according to: effect on protein and transcript; patient phenotype (www.human-phenotype-ontology.org); inheritance model; minor allele frequency in general population. Candidate variants – classified according to ACMG guidelines [(2)] – should have a consistent pathogenic mechanism. Pathogenic variants were confirmed in the proband and parents by Sanger sequencing using a second independent DNA sample.

Pathology studies

For optical microscopy, liver tissue underwent standard hematoxylin/eosin and Masson trichrome stains.

For transmission electron microscopy, after fixation, inclusion, and toluidine/sodium tetraborate staining of semithin sections, blocks were thin-sectioned at 85 nm, and stained in saturated uranyl acetate in 99% alcohol and Reynold's lead citrate.

RNA studies

Upon consent, liver tissue was collected from hepatectomy in the patient and in three age-matched children undergoing liver transplant (LT) for biliary atresia (BA), and from an age-matched donor following whole LT, and subjected to gene expression analysis using QuantSeq 3'-mRNA sequencing. RNA extraction, amplification and cDNA sequencing were performed as previously described [(3)]. The sequence reads were trimmed using the Trim Galore software (https://www.bioinformatics.babraham.ac.uk/projects/trim_galore/), and aligned on the hg19 reference sequence using STAR. Genes expression was

determined with htseq-count using the Gencode v19 gene model. Differential expression analysis was performed using EdgeR. A false discovery rate (FDR) < 0.05 was used as a threshold.

WD-related human homologue genes were extrapolated from a genome-wide mRNA profiling in *ATP7B*-deficient mice [(4)].

For gene ontology enrichment analysis, genes significantly up- or down-regulated at least 2-fold were clustered according to the Kyoto Encyclopedia of Genes and Genomes (www.genome.jp/kegg) and compared with three system biology analysis in rodent models of WD [(5)].

References

1. Nicastro E, Di Giorgio A, Marchetti D, et al. Diagnostic Yield of an Algorithm for Neonatal and Infantile Cholestasis Integrating Next-Generation Sequencing. J Pediatr 2019; 211:54-62.e4. https://doi.org/10.1016/j.jpeds.2019.04.016

2. Nykamp K, Anderson M, Powers M, et al. Sherloc: a comprehensive refinement of the ACMG-AMP variant classification criteria. Genet Med Off J Am Coll Med Genet 2017; 19:1105–1117. https://doi.org/10.1038/gim.2017.37

3. Polishchuk EV, Merolla A, Lichtmannegger J, et al. Activation of Autophagy, Observed in Liver Tissues From Patients With Wilson Disease and From ATP7B-Deficient Animals, Protects Hepatocytes From Copper-Induced Apoptosis. Gastroenterology 2019; 156:1173-1189.e5. https://doi.org/10.1053/j.gastro.2018.11.032

4. Huster D, Purnat TD, Burkhead JL, et al. High copper selectively alters lipid metabolism and cell cycle machinery in the mouse model of Wilson disease. J Biol Chem 2007; 282:8343–8355. https://doi.org/10.1074/jbc.M607496200

5. Burkhead JL, Gray LW, Lutsenko S. Systems biology approach to Wilson's disease. Biometals Int J Role Met Ions Biol Biochem Med 2011; 24:455–466. https://doi.org/10.1007/s10534-011-9430-9
